# Association of low serum 25-hydroxyvitamin D levels with hearing loss severity in Meniere disease: a cross-sectional study

**DOI:** 10.3389/fneur.2025.1638357

**Published:** 2025-09-01

**Authors:** Yunqin Wu, Zhenzhen Lai, Ange Li, Weiwei Han, Xiaoxia Liu, Weinv Fan

**Affiliations:** ^1^Department of Neurology, Ningbo No. 2 Hospital, Ningbo, China; ^2^Department of Neurology, Tiantai People’s Hospital of Zhejiang Provine, Tiantai Branch of Zhejiang Provincial People’s Hospital, Hangzhou Medical College, Taizhou, Zhejiang, China; ^3^School of Medicine, Shaoxing University, Shaoxing, China; ^4^Department of Rehabilitation, Ningbo No.2 Hospital, Ningbo, China

**Keywords:** hearing loss, Meniere disease, otolithic membrane, vitamin D, vitamin D deficiency

## Abstract

**Introduction:**

Growing evidence implicates vitamin D in inner ear homeostasis, though its association with Ménière’s disease (MD) remains incompletely characterized. This study aimed to compare serum 25-hydroxyvitamin D (25(OH)D) levels between patients with MD and healthy controls and to assess its correlation with hearing thresholds in MD cohort.

**Methods:**

In this cross-sectional study, 49 patients with MD and 250 matched healthy controls were enrolled at our institution between January 2023 and January 2025. Groups were matched for demographics, comorbidities, exercise habits, and seasonal blood collection timing. Serum 25(OH)D levels and pure-tone audiometry (PTA) results were measured and analyzed.

**Results:**

Patients with MD exhibited significantly lower 25(OH)D levels than controls [18.4 ± 5.9 (45.9 ± 14.7 nmol/L) vs. 21.1 ± 6.2 ng/mL (52.7 ± 15.5 nmol/L); *p* = 0.006]. After adjusting for covariates, vitamin D deficiency was independently associated with MD (adjusted OR = 2.21; 95% CI: 1.13–4.32; *p* = 0.021). A moderate inverse correlation existed between 25(OH)D and PTA thresholds (*ρ* = −0.440, *p* = 0.002).

**Conclusion:**

Hypovitaminosis D is associated with MD and hearing loss severity, warranting further longitudinal studies to explore causality and therapeutic implications.

## 1 Introduction

Meniere’s disease (MD) remains a challenging clinical entity characterized by the triad of recurrent vertigo, fluctuating hearing loss, and tinnitus, often accompanied by aural fullness (1). Although endolymphatic hydrops represents the pathological hallmark, its precise etiopathogenesis involves multifactorial interactions, including autoimmune dysregulation, genetic predisposition, viral infection, and neurovascular compromise. Of particular interest, displaced otoconia may disrupt inner ear homeostasis, potentially triggering MD symptoms (2, 3). Current therapeutic options for MD remain limited and include sodium restriction, intratympanic gentamicin or steroids administration, and endolymphatic sac decompression (3).

Vitamin D modulates immunity and vascular integrity beyond calcium regulation (4). Notably, vitamin D receptors (VDRs) are abundantly expressed in the inner ear, particularly in hair cells, spiral ganglion neurons, and stria vascularis. VDR-knockout (VDR−/−) mice develop hearing loss and vestibular dysfunction, with histopathological evidence of hair cell apoptosis and otoconia demineralization (5). Clinical studies link vitamin D deficiency to higher recurrence rates in benign paroxysmal positional vertigo (BPPV), as well as poorer recovery outcomes in idiopathic sudden sensorineural hearing loss (SSNHL) (6, 7).

Prior study by Bakhshaee et al. (8), first demonstrated significantly lower serum 25-hydroxyvitamin D (25(OH)D) levels in patients with MD compared to matched controls, but did not examine links to symptom severity/frequency or therapeutic implications. While Buki et al. (9) theorized vitamin D deficiency might exacerbate MD symptoms via immune dysregulation or otolithic instability, their suggested symptomatic benefits of supplementation relied solely on unvalidated clinical observations. Consequently, direct evidence linking vitamin D status to MD phenotype remains limited.

Given the anatomical concordance between vitamin D target tissues and sites of MD pathology, we hypothesized that serum 25(OH)D levels might be associated with MD. This study was designed to address this knowledge gap with two primary objectives: (1) to compare 25(OH)D status between patients with MD and healthy controls, and (2) to evaluate potential dose–response relationships between 25(OH)D concentrations and auditory parameters.

## 2 Methods

### 2.1 Participants selection and study design

This cross-sectional study was approved by the Institutional Review Board of Ningbo No.2 Hospital (Protocol KY-2023-119; approved 9 January 2023) and conducted in accordance with the World Medical Association’s Declaration of Helsinki. Written consents were obtained from all participants.

We consecutively recruited patients with definite MD, diagnosed according to American Academy of Otolaryngology-Head and Neck Surgery (AAO-HNS) criteria (10), from the Department of Neurology and Otorhinolaryngology between January 2023 and January 2025. To confirm the diagnosis, some patients underwent ancillary tests, such as ocular and cervical vestibular evoked myogenic potentials (o/c VEMP), video head impulse test, brainstem evoked response audiometry, and imaging. Controls were frequency-matched 1:5 to MD cases by age, sex, enrollment season (defined per Ningbo’s climate as spring: Mar-May, summer: Jun-Aug, autumn: Sep-Nov, winter: Dec-Feb), and smoking/drinking status (current/former/never). All participants completed standardized questionnaires covering demographics, medical history (BMI, comorbidities, medications), sun exposure habits [daily outdoor duration (hours); sunscreen use frequency (never, rarely, sometimes, often, always)], and physical activity patterns (≥150 min/week).

These exclusion criteria for all participants were as follows: (1) systemic or chronic serious disease affecting vitamin D metabolism, such as chronic kidney/liver disease, bone metabolism disorders, gastrointestinal disorders, hormonal disorders, or connective tissue disease; (2) current use of vitamin D supplements or corticosteroids; (3) incomplete baseline data or inability to comply with study procedures.

### 2.2 Measurement of 25(OH)D concentration

Fasting morning venous blood samples were collected from all participants across all seasons to minimize diurnal and seasonal variability. Serum 25(OH)D-the preferred biomarker due to its prolonged half-life (2–3 weeks) and superior stability compared to the active metabolite 1,25-dihydroxyvitamin D. Serum 25(OH)D was quantified via liquid chromatography–tandem mass spectrometry (11) and categorized per Endocrine Society guidelines: deficiency (<20 ng/mL [<50 nmol/L]), insufficiency (20–29 ng/mL [50–74 nmol/L]), or sufficiency (≥30 ng/mL [≥75 nmol/L]) (12).

### 2.3 Pure-tone audiometry testing (PTA)

All patients underwent a PTA using a clinical audiometer with TDH 39 headphones and B-71 bone vibrators for air and bone conduction, respectively. Hearing thresholds were tested bilaterally at octave frequencies of 125 Hz to 8 kHz in a sound-attenuated booth meeting ISO 8253-1 specifications. Clinical stage was staged per AAO-HNS criteria using PTA: Stage 1: ≤25 dB HL; Stage 2: 26–40 dB HL; Stage 3: 41–70 dB HL; Stage 4: PTA > 70 dB HL (10). Bilateral cases were classified by the worse-hearing ear.

### 2.4 Statistical analysis

Data analysis was conducted using SPSS 22.0. Continuous variables were presented as means ± SD (normally distributed) or median (IQR; non-normal), assessed via Kolmogorov–Smirnov test. Categorical variables were described as numbers (percentages). Group comparisons used t-test, chi-square test, or Mann–Whitney U test, as appropriate. Multiple logistic regression and Spearman correlation test assessed adjusted associations. *p* < 0.05 was considered statistical significance.

## 3 Results

### 3.1 Demographic and characteristic data of MD patients and healthy controls

From an initial screening of 67 potential participants, 49 patients with MD meeting the inclusion criteria were enrolled. Exclusions comprised unavailable vitamin D assays (*n* = 9), incomplete baseline data (*n* = 5), and current use of vitamin D -affecting medications (*n* = 4). The final MD cohort (mean age 59.4 ± 12.8 years; 67.3% female) exhibited similar comorbidity profiles to healthy controls (*n* = 250), with hypertension prevalence at 28.6% and diabetes mellitus at 10.2%. Neither seasonal distribution nor regular exercise differ significantly between groups (all *p* > 0.05; Table 1).

**Table 1:** Demographic characteristics of patients with MD and healthy controls.

Characteristics	MD (*n* = 49)	Healthy control (*n* = 250)	*p*-value
Sex (F/M)	33/16	169/81	0.972
Age (year)	59.4 ± 12.8	61.5 ± 11.7	0.243
BMI (kg/m^2^)	24.3 ± 3.4	23.6 ± 3.2	0.191
Smoking [*n* (%)]	13 (26.5%)	79(31.6%)	0.482
Drinking [*n* (%)]	8 (16.3%)	51(20.4%)	0.512
Diabetes [*n* (%)]	5 (10.2%)	46(18.4%)	0.163
Hypertension [*n* (%)]	14 (28.6%)	92(36.8%)	0.271
Regular exercise	11 (22.4%)	72 (28.8%)	0.364
Spring	13(26.5%)	62(24.8%)	0.737
Summer	12(24.5%)	59(23.6%)	
Autumn	15(30.6%)	65(26.0%)	
Winter	9(18.4%)	64(25.6%)	
25(OH)D (ng/mL)	18.4 ± 5.9	21.1 ± 6.2	0.006

MD, Meniere’s disease; Values are expressed as *n* (%), mean ±SD or median (interquartile range). Chi-squared test, *T*-test, were used to generate *p* values for variables. *p*-values < 0.05 were considered significant.

**Figure 1:**
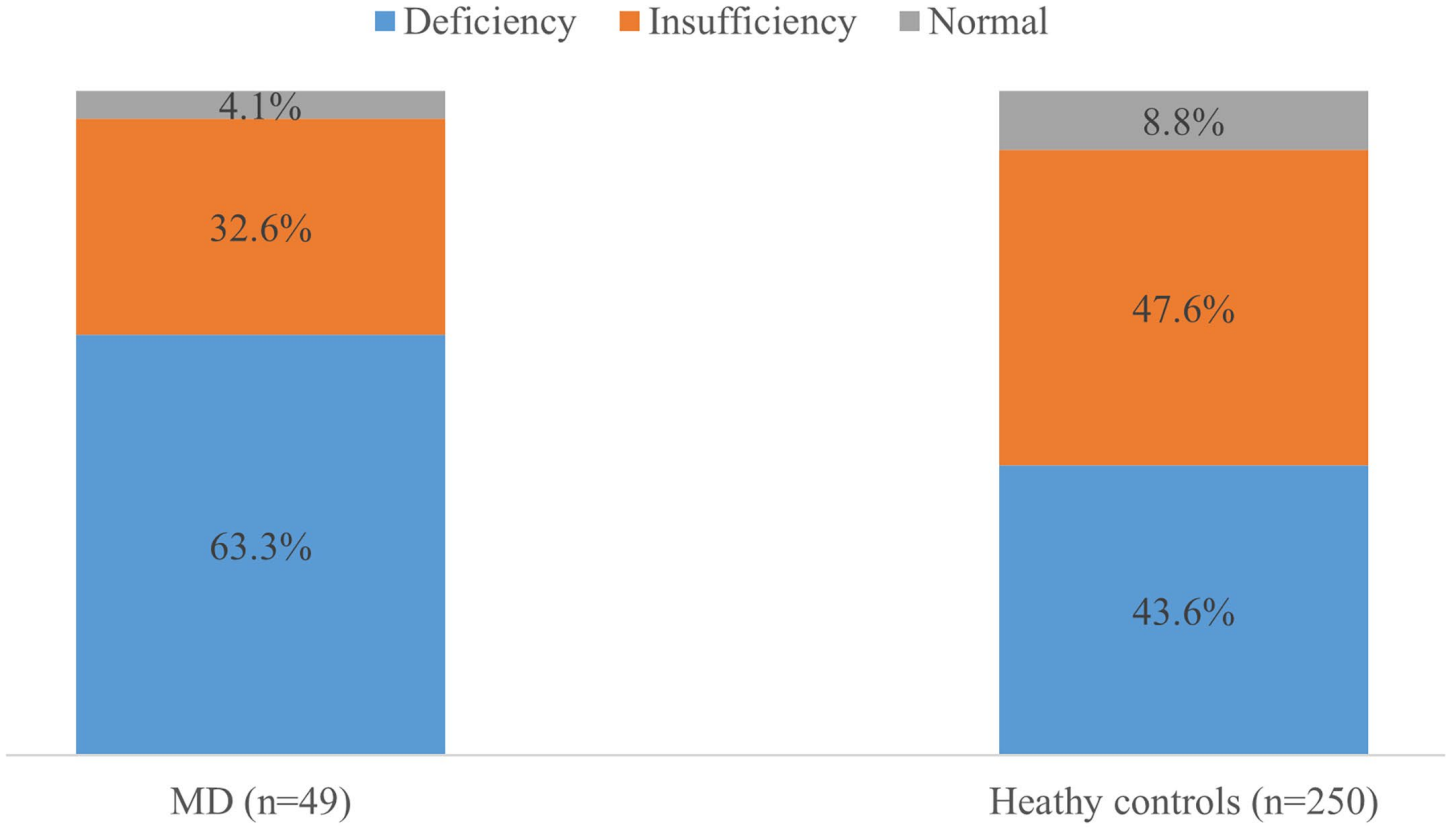
Prevalence of vitamin D status in the patients and healthy controls. The proportion of vitamin D deficiency (<20 ng/mL) was higher in patients with Meniere’s disease (MD) than in healthy controls (*p* = 0.06).

Serum 25(OH)D concentrations were significantly lower in patients with MD (18.4 ± 5.9 ng/mL [45.9 ± 14.7 nmol/L]) compared to healthy controls (21.1 ± 6.2 ng/mL [52.7 ± 15.5 nmol/L]; *p* = 0.006). Vitamin D deficiency was observed in 63.3% (*n* = 31) of MD patients, 32.6% (*n* = 16) exhibited insufficiency and 4.1% (*n* = 2) had sufficient levels. In controls, these proportions were 43.6, 47.6, and 8.8%, respectively (Figure 1). Multivariate logistic regression adjusted for age, sex, BMI, diabetes, hypertension, regular exercise, and season revealed an independent association between vitamin D deficiency and MD (adjusted OR = 2.21; 95% CI: 1.13–4.32; *p* = 0.021).

### 3.2 Association between vitamin D levels and hearing impairment

When stratified by low-frequency hearing loss severity (500–2000 Hz mean thresholds), the MD cohort included 20 stage 1, 15 stage 2, 8 stage 3, and 6 stage 4 cases. Serum 25(OH)D levels showed a significant inverse correlation with hearing impairment severity (Spearman’s *ρ* = −0.440; *p* = 0.002), which remained significant after adjusting for confounding variables (adjusted *ρ* = −0.388; *p* = 0.008).

## 4 Discussion

Serum 25(OH)D in patients with MD were significantly lower than matched controls (*p* = 0.006). Notably, 63.3% of MD patients met criteria for vitamin D deficiency, a prevalence 1.45-fold higher than in controls (43.6%). Moreover, a dose-dependent inverse correlation existed between 25(OH)D and hearing thresholds (*ρ* = −0.440, *p* = 0.002), suggesting a potential dose–response relationship. These findings align with emerging evidence linking hypovitaminosis D to inner ear disorders, including BPPV and SSNHL. While prior studies have implicated vitamin D in otolith metabolism and cochlear function, our study provides the first clinical evidence of its association with both MD prevalence and auditory dysfunction severity in a well-phenotype cohort.

Accumulating clinical evidence demonstrates a consistent association between hypovitaminosis D and various otologic conditions. Epidemiological studies have established that patients with BPPV, vestibular neuritis (VN), and SSNHL exhibit significantly reduced serum 25(OH)D concentrations compared to healthy controls. Importantly, correcting vitamin D deficiency was beneficial for alleviating clinical symptoms and reducing BPPV recurrence by 40% in randomized controlled trial (7, 13, 14), and for reducing high-frequency hearing loss risk in SSNHL. Objective vestibular testing demonstrates that vitamin D deficiency significantly alters otolith-vestibular reflexes, with abnormal oVEMP responses observed in 68% of deficient individuals versus only 22% of those with sufficient levels (15).

While these findings strongly implicate vitamin D in peripheral vestibular function, its specific role in MD remains understudied. Preliminary investigation by Bakhshaee et al. (8) in a small MD cohort (*n* = 28) reported serum 25(OH)D levels lower than controls (15.2 vs. 19.8 ng/mL, *p* = 0.03). Critically, our finding advances prior work by establishing a novel inverse correlation between serum 25(OH)D levels and objective hearing loss severity (*ρ* = −0.440, *p* = 0.002). This dose-dependent relationship with audiometric outcomes distinct from earlier studies linking vitamin D solely to MD prevalence. More compellingly, a prospective intervention showed 55% lower intratympanic gentamicin use in MD patients with supplementation vitamin D (9).

Vitamin D levels are influenced by multiple factors, such as season, geographic location, skin color, lifestyle, supplement use, nutritional status, and measurement methodology (16). To mitigate seasonal and geographic effects, healthy controls were recruited from our hospital’s health check-up center at a 1:5 ratio to cases, matched by local community and recruitment timeframe. We also compared potential confounders (age, sex, BMI, comorbidities) between groups. Furthermore, the mean vitamin D level (21.1 ± 6.2 ng/mL) and frequency of deficiency (43.6%) in our control group closely mirrored the Zhejiang Province epidemiological survey values (21.26 ± 7.72 ng/mL and 48.2%, respectively) (17). These methodological measures strengthen the reliability of our findings.

Physiologically, endolymphatic calcium concentrations (~20–30 μM) are critical for normal hair cell function. It proposes that vitamin D deficiency elevates endolymphatic Ca^2+^ via three putative mechanisms: (1) dissolution of displaced otoconia; (2) impaired calcium buffering by vitamin D-dependent binding proteins; and (3) dysregulated VDR-mediated ion channel function. Resultant calcium overload disrupts stereocilia transduction and promotes endolymphatic hydrops via ductus reuniens obstruction by otolithic debris (3, 5, 18). VDR−/− mice exhibit a pathological triad: disorganized otoconia mineralization, cochlear-vestibular ganglion degeneration, and premature hair cell loss with stereocilia defects (4, 19). Collectively, these findings implicate vitamin D deficiency as a potential predisposing factor for MD, simultaneously affecting labyrinthine structures, the otic capsule, and neural elements. The confluence of these pathologies likely contributes to the characteristic MD symptomatology (1, 20).

Growing evidence implicates viral triggers (e.g., HSV-1 DNA detected in MD temporal bones) and immune dysregulation in MD pathogenesis, characterized by sensory neuron invasion, otolithic membrane degeneration, and perivascular inflammation (21, 22). These processes disrupt the blood-labyrinth barrier via cytokine-mediated increases in vascular permeability (TNF-*α*, IL-6) (23). Vitamin D exhibits multimodal immunomodulation, downregulating NF-κB signaling, suppressing pro-inflammatory cytokine production, and stabilizing endothelial junctions (5, 24, 25), potentially counteracting these effects. Genetic studies further identify vitamin-D-responsive risk alleles affecting cell adhesion molecules (e.g., PCDH15) in MD. Critically, emerging immunophenotyping classifies MD into: autoinflammatory (13%, NLRP3 inflammasome activation, elevated IL-1β), autoimmune (20%, high TNF-*α*), allergic (25%, type 2 cytokines, IgE), and low cytokine levels (42%) (26). Vitamin D exerts subtype-specific immunomodulation: suppressing NLRP3 inflammasome activation and IL-1β maturation (relevant to autoinflammatory subtype); downregulating Th2 responses, IgE production, and mast cell degranulation (relevant to allergic subtype); and enhancing Treg function and attenuates TNF-*α*-mediated inflammation (relevant to autoimmune subtype) (5, 27). Clinically, adequate serum 25(OH)D correlates with attenuated inner ear inflammation and preserved barrier integrity (28, 29). Thus, deficiency may permit unchecked immune-mediated damage across immunophenotypes, highlighting its therapeutic potential-particularly in IL-1β/IgE-dominant subtypes.

This study has several limitations. First, the cross-sectional design precludes causal inference regarding serum vitamin D levels and MD. Second, residual confounding may persist despite covariate adjustment, including unquantified dietary vitamin D/calcium intake, UV exposure patterns, sunscreen use, and genetic variants VDR/CYP2R1 /GC polymorphisms, which were hard to control. Third, biological interpretability is constrained by: (a) lack of objective audiometry in controls, and (b) absence of serum calcium, phosphate, and parathyroid hormone measurements. Finally, although described as well-phenotype regarding core diagnostic criteria, key clinical variables: including disease duration, vertigo attack frequency, bilateral involvement, and vestibular suppressant use-were not analyzed. These factors may potentially influence both vitamin D status (through behavioral modifications like reduced outdoor activity) and hearing outcomes. While the difference in mean 25(OH)D levels between MD patients and controls (2.7 ng/mL) is modest, its clinical relevance is underscored by the strong independent association of vitamin D deficiency with MD (adjusted OR = 2.21) and significant inverse correlation with hearing loss severity (*ρ* = −0.440). Future multicenter studies with expanded cohorts should integrate genetic profiling, lifestyle factors, advanced imaging, and animal models to elucidate the mechanistic links between vitamin D metabolism and the pathogenesis of MD.

## 5 Conclusion

This cross-sectional study demonstrates a significant association between vitamin D deficiency and both MD prevalence and hearing loss severity, suggesting that hypovitaminosis D may associated with MD pathogenesis.

## Data Availability

The raw data supporting the conclusions of this article will be made available by the authors, without undue reservation.
